# COVID-19 Vaccine Refusal and Medical Distrust Held by Correctional Officers

**DOI:** 10.3390/vaccines11071237

**Published:** 2023-07-13

**Authors:** Erin Michelle Turner Kerrison, Jordan M. Hyatt

**Affiliations:** 1 School of Social Welfare, University of California, 120 Haviland Hall #7400, Berkeley, CA 94720, USA; 2 College of Arts and Sciences, Drexel University, Philadelphia, PA 19104, USA

**Keywords:** COVID-19, incarceration, legal epidemiology, medical distrust, occupational health, prison, structural competency, vaccine hesitancy, vaccine uptake

## Abstract

This study explores COVID-19 vaccine acceptance among prison security staff and the extent to which they trust varied sources of information about the vaccines. Cross-sectional survey data were obtained from a state-wide sample of corrections officers (COs, hereafter; n = 1208) in February 2021. Group differences, disaggregated by demographic characteristics, were examined using F-tests and *t*-tests. Despite the comparatively limited risk of contracting the virus, non-security staff reported they would accept a COVID-19 vaccine at no cost (74%), compared to their more vulnerable CO counterparts (49%). We observed vaccine refusal correlations between COs’ reported gender, age, and length of time working as a CO, but none with their self-reported race. Vaccine refusal was more prevalent among womxn officers, younger officers, and those who had spent less time working as prison security staff. Our findings also suggest that the only trusted source of information about vaccines were family members and only for officers who would refuse the vaccine; the quality of trust placed in those sources, however, was not substantially positive and did not vary greatly across CO racial groups. By highlighting characteristics of the observed gaps in COVID-19 vaccine acceptance between COs and their non-security staff coworkers, as well as between corrections officers of varied demographic backgrounds, these findings can inform the development of responsive and accepted occupational health policies for communities both inside and intrinsically linked to prisons.

## 1. Introduction

Despite witnessing a global pandemic and the largest global vaccination campaign in history, including guidance surrounding COVID-19 exposure risk and evidence-based preventative measures, many people still hold reservations about the safety of the vaccine for the novel coronavirus, perhaps especially so when they hold diminished faith in the campaign’s public and private architects. It is unsurprising that a lack of trust in the government, public health officials, and scientists, writ large, could reinforce racial disparities in state-authored COVID-19 vaccine protocol buy-in [1,2,3]. Among socioeconomically marginalized communities in the US, the reasons to doubt a state-sponsored health campaign are manifold, including the forced sterilization of Indigenous communities [4], asylum commitment of those deemed disabled by immigration authorities [5,6], and the intentional spread of syphilis among Black Americans [7,8]. Even when reporting poorer health, the chasm between the general public’s trust and a willingness to access its public health systems is particularly wide for ethnoracially marginalized people who have historically contended with state-sponsored violence [9,10,11,12]. Emergent research suggests that despite their acute COVID-19 exposure risk, vaccine opposition is particularly endorsed within the US first responder workforce (e.g., police officers, fire fighters, and emergency medical service providers) [13,14,15]. Our study explores observable differences between prison security enforcement staff (i.e., corrections officers) vaccine uptake and their non-security staff counterparts, the extent to which officers’ self-reported demographics are correlated with vaccine uptake, and the extent to which self-reported demographic details correlate with their trust in sources of information about the vaccines. 

Corrections officers (COs, hereafter) are designated public safety essential workers whose occupational health is already threatened by working in a fundamentally dangerous environment. Job duties include preventing escape, confiscating contraband materials, recording inmate movement, identifying inmates’ mental and behavioral aberrations, deploying emergency and crisis response protocols, and maintaining operational order, in general–resultant job dissatisfaction and burnout are well documented [16,17,18]. Their exposure to and attitudes about COVID-19, however, is a nascent line of inquiry that merits closer attention from the occupational health, environmental medical community, medical sociology, sociolegal scholarly communities, among others. This is especially true for studying staff whose corrections system employers encourage vaccination against COVID-19 but have not formally designed a mandatory vaccination protocol. 

Officers face exposure to COVID-19 and other lethal viruses by virtue of their proximity to detained populations where communicable respiratory disease can spread quickly and by their proximity to populations beyond the prison walls were neither vaccinated nor practicing social distancing or face-masking protocols. In addition to these COVID-19 exposure risks, study findings published by the US Department of Justice suggest that among the dangers and risks that COs confront, diminished trust in the prison system leadership’s commitment to ensuring their health and physical safety remains a longstanding issue [19,20]. Thus, across correctional systems in the US, convincing corrections staff to voluntarily accept the COVID-19 vaccine has been challenging [21,22].

Evidence suggests that *medical distrust* [23,24,25,26]–a sustained skepticism about a healthcare provider’s intentions, trustworthiness, and competence–is common among non-White individuals [27,28] and positively associated with poor health [29]. Measuring distrust of healthcare providers for a prison-based sample is critical as this health landscape is particularly bleak; a disproportionate number of detainees in US prisons are Black and Latinx and these incarcerated adults are generally less healthy than non-incarcerated adults [30]. Within CO populations, sleep disorders [31], substance misuse [17], physical injuries [32], and suicidality [33] are all common. Corrections officers are charged with maintaining order and safety in these facilities, which can compound endemic distrust and the myriad work-related poor occupational health outcomes that they confront.

Recent findings show that increasing prison-based mortality rates driven solely by COVID-19 outpace the COVID-19-related life expectancy declines observed in the general population [34,35]. In both state and federal prisons, relative to the general US population, prison staff experience a much higher prevalence of COVID-19 cases [36]. While prison administration must provide vaccines for the individuals that move through these spaces, at least two challenges rooted in distrust can be attributed to why vaccine hesitancy poses a serious threat to sustainable system-wide vaccination efforts. 

First, prison-based healthcare provision is generally ill-equipped to manage the longstanding and compounded threats to health when maintaining security is prioritized above delivering higher standards of medical care. Simultaneously, asymmetrical authority and limited personal agency arrangements undermine the consent processes required for ethical medical intervention [37]. Physicians who can tackle these challenges are difficult to recruit due in part to concerns about career advancement, burnout, and job satisfaction [38]. Finally, the history of carceral systems failing to deliver an equivalence of care [39] and engaging in experimentation on vulnerable subjects [40,41] may not instill confidence in the current medical establishment, neither for incarcerated adults nor the frontline security staff who bear witness to those realities. 

Second, US prisons are disproportionately populated by Black men who have many reasons to distrust state agents [9,28,40,42]. Recent estimates report that more than 40% of incarcerated adults are Black men [43], they come to custody with poorer health status (relative to men of other ethnoracial categories) [30], have likely confronted routine criminal legal system intrusion as the most policing-surveilled subjects in the US [10,44,45,46], and they are the largest proportion of US residents who are lethally brutalized by police [47]. At the same time, many prisons are staffed by Black men who come from these very same policed communities and are drawn to prison work because of the financial incentives that they could not obtain in other labor market sectors [48]. Little empirical research has been established about Black prison staff’s positions on healthcare utilization, particularly while navigating additional COVID-19-related challenges [49,50]. 

In addition to the empirical work discussed below, we invite readers to engage with this study in tandem with two significant conceptual contributions to the discourse on medical distrust. First, to examine how these surveillance systems dampen policed people’s reliance on public healthcare [10] we turn to *legal epidemiology*, or an analytic framework for understanding how law, state-governed public agencies, and legal system practices condition structural determinants of health [10]. Legal epidemiology offers a context for exploring how systemic medical racism [51] emerges in discrete and overlapping state-sanctioned structures, including prison health systems. Second, we build on existing literature that explores the scope of *place-based medical distrust* [10,47,52,53,54]–prison in this study [55,56,57,58]–by defining the perceptions of safety held by prison security staff who work in one of the largest state correctional systems in the US. Specifically, we define and measure the prevalence of COVID-19 vaccination hesitancy for this understudied ethnoracially diverse group of workers to generate insights into which influential and trusted sources of health-related information may inform vulnerable officers’ willingness to accept a COVID-19 vaccine administered by the prison in which they work.

Our dual hypotheses are as follows: (1) relative to their counterparts, Black COs will be overrepresented among respondents who would refuse the vaccine; and (2) relative to their counterparts, Black COs will be the least trustful of prison-based healthcare providers. 

## 2. Materials and Methods

The Pennsylvania Department of Corrections (PADOC, hereafter) includes 23 State Correctional Institutions (SCIs), one motivational boot camp, 14 state-run and over 40 privately contracted community-based facilities, and employs over 15,000 people [59]. The PADOC maintains one of the top ten highest prison populations in the US and at the conclusion of March 2021, around when the survey data were collected (procedures detailed below), the total residential population was 38,262 individuals [60].

### 2.1. Survey Instrument

In December 2020, the research team began hosting regularly scheduled meetings with PADOC leadership to better understand their policy concerns, critical issues regarding health, safety, and vaccination within the SCI context, as well as their nascent plans for vaccinating carceral populations when a vaccine became available. After conducting a pilot survey with the medical and management leadership, several rounds of revisions, as well as addressing written comments from the medical, administrative, and research personnel leads within the PADOC, the two tailored surveys were finalized: one version for corrections staff and one version for incarcerated people, each of which is described below. Due to the mandated population lockdowns, however, the survey instrument could not be piloted among the incarcerated sample.

The pandemic, and the urgent safety protocols in place within the SCIs, required the development of new methods of surveying carceral populations. For the incarcerated respondents, the first implementation plan and administration protocol were developed with an emphasis on the use of the kiosks located on each housing unit. Concerns about sanitation, congregation in a single location, and overuse of the kiosk resulted in the development of a revised plan. Under this plan, tablet computers were provided to each facility for the completion of the survey.

To understand both the incarcerated and officer populations’ beliefs about the global pandemic, a unique survey instrument was developed by PADOC leadership in consultation with both authors of this study. The 41-question survey used Likert-style items to allow detainees and staff to self-report their beliefs and opinions in several areas relevant to vaccination and corrections policy. While data analyses for the incarcerated sample’s survey responses are beyond the scope of this manuscript’s aims, we offer a brief explanation of how we ensured their access to the survey. 

First, a stratified random sample of incarcerated people (n = 2011) from across all SCIs was identified by the Office of Planning, Research, and Statistics. This approach ensured that all facilities would be represented within the final dataset and that the distribution of responses would mirror, to the extent possible, the population of the PADOC. The survey was loaded onto tablet computers that were to be used exclusively for this particular data collection effort. These 47 tablets were mailed to each SCI on 5 February 2021. Business office staff under the supervision of the Office of Administration in each SCI received the tablet in the mail along with the names of the individuals previously selected for participation (note that the research team does not have access to any participant identifiers). Beginning on 8 February 2021, the staff member located the incarcerated person and provided them the opportunity to participate. Completing the survey was voluntary, though incarcerated people who did so were compensated for their time in the form of receiving outgoing email credits.

Identical procedures were followed at each of the SCIs, with limited variation allowed to accommodate for differences in security levels and other site-specific characteristics. At all times during data collection, PADOC and Centers for Disease Control COVID-19 guidelines were to be followed by all parties (e.g., sanitization of tablets, social distancing, and wearing face masks). On 19 February 2021, the data collection period concluded. At that time, all 47 tablets were returned to Central Office, the data were deidentified by PADOC staff and provided to the outside research team for analysis. Overall, 1498 complete or partial responses were received (response rate: 76.3%).

The procedures used to obtain data from the corrections staff population were more straightforward. A unique survey access link was sent to all active PADOC official staff email accounts (N = approximately 15,000) on 29 January 2021. The weblink remained active until 12 February 2021, a period of 14 days. The PADOC did not send follow-up or reminder emails, nor were any incentives provided for participation. At the conclusion of the data collection period, 4232 complete or partial responses were received. This represents an overall response rate of approximately 28.2%, a rate largely on par with non-incentivized and anonymous private health-related web surveys of this nature [61]. 

When the surveys were administered in January and February od 2021 there were no COVID-19 vaccines approved for use in the United States. Since that time, three manufacturers have obtained Food and Drug Administration (FDA) approval to supply vaccines for domestic use (Pfizer, Moderna, and Johnson & Johnson). Under an “emergency use authorization” approval from the FDA [62], incarcerated people and PADOC staff were able to receive a vaccination at no cost beginning in approximately March of 2021. Receipt of a vaccine, at that time, was voluntary. In August of 2021, after the FDA finalized a complete regulatory review and gave the Pfizer-produced coronavirus vaccine full approval, then Pennsylvania Governor, Tom Wolf, mandated that all state employees working in congregate-care facilities (which would include all SCIs) be fully vaccinated against COVID-19 by 7 September 2021. Though PADOC staff are not required to report protected health information to their employer, as of July 2023, 44.8% of all correctional staff self-reported that they were fully vaccinated [63]. 

Several related studies that measure COVID vaccination trends in US carceral settings rely on electronic medical records of incarcerated adults [22], state and federal prison-hosted COVID-tracking datasets [64,65], as well as semi-structured interviews with Cos [50,66], incarcerated adults [67], and both stakeholder groups at the same facility [68]. Due to necessity and buoyed by the strong research partnership we have cultivated with the PADOC and other criminal justice agencies [69,70], the survey administration design and protocol described above are unlike any others to date. In addition to measuring attitudes about COVID-19 transmission and vaccination, we have established a new model and standard for survey design and administration in carceral sites that are subject to highly transmissible and potentially fatal diseases.

### 2.2. Study Sample

In early 2021 before any COVID-19 vaccines were available, staff received the survey from the PADOC at via their official PADOC email accounts. Out of approximately 15,000 eligible and invited staff respondents (i.e., all full-time PADOC employees across all prison facilities and offices), we recorded 4232 responses (response rate: 28.2%). Given our focus on the perceptions of PADOC staff who routinely share most of their time in proximity with those in custody, our analyses of staff attitudes are tailored in two important ways. The analyses below include responses from the 1208 people who both identified as prison security staff based on information provided (i.e., those who identified as an “officer”, “guard”, security-related “sergeant”, etc.) and who answered the question of whether they would accept a COVID-19 vaccine. All survey data were self-reported and each prison facility was represented. We restricted the analyses to CO participants who identified as Black, Hispanic, and White and those who indicated if they would take the vaccine. Due to their small numbers and resultant identifiability, as well as the unreliability of yielding useful analytic results about each ethnoracial subgroup, staff who reported they were “American Indian or Alaska Native” (n = 18) and “Another Race” (n = 106) were excluded from the calculations reported below.

### 2.3. Measures

Related surveys designed to measure medical distrust have focused largely on the perceptions of minoritized ethnoracial medical patients [71,72] and the biased perceptions of physicians charged with their care [73]. Closer to our study focus are surveys administered to incarcerated adults who rely on jail-based [56,74] or prison-administered [56] healthcare. To our knowledge, however, ours is the first survey to capture prison staff perceptions of work-place healthcare provision during a global pandemic for which the vaccination rollout was considered too rushed by many who questioned its safety [56,75]. Though survey research methods were not used in all related studies, see Ferdik et al.’s 2022 [66] analyses of semi-structured interview data from COs (n = 21) and Martin-Howard’s (2022) [50] qualitative study with Rikers Island COs (n = 15). Despite reviewing those survey designs, we created our own novel instrument because we are focused on a vastly understudied employee population during a moment of heightened distrust and concern about the state-sponsored sources of the vaccines and their administration. 

PADOC personnel were invited to complete the 41-item survey asking respondents to self-report their opinions and agreement with beliefs across five primary foci: general vaccine efficacy, individual vaccination history, trust in prison medical staff, perceived COVID-19 exposure risk, and COVID-19 vaccine safety. The two primary study outcomes of interest are (1) corrections officers’ acceptance of the COVID-19 vaccine and (2) the information sources that COs believe are informed enough to offer truthful information about COVID-19 vaccine safety. Likert-style survey items (ranging from 1 = strongly disagree to 5 = strongly agree) and binary survey items (ranging from 0 = no to 1 = yes) asked participants to indicate their willingness to accept a cost-free vaccine for COVID-19, as well as to evaluate a series of statements regarding vaccination history, perceived risk of contracting COVID-19 and trust in medical providers. These statements were collapsed into scales that measured the following five constructs:

- Belief in vaccines to prevent contracting a serious disease
- Prior exposure to vaccinations
- Trust in medical providers
- Perceived COVID-19 exposure risk
- Beliefs about COVID-19 vaccine safety (specifically)

Some items were reverse coded to ensure that higher scores align substantively across variables. Respondents were also asked to agree or disagree with statements about who they trust regarding information about vaccines. Response options included medical providers, government, PADOC leadership, family, external community leaders, and other incarcerated people (for the incarcerated sample’s survey instrument) or prison staff (for the CO sample’s survey instrument). In addition, respondents were asked a series of questions eliciting basic demographic information such as their age, gender, race, ethnicity and tenure with the PADOC. 

## 3. Statistical Analysis 

We analyze the data descriptively, focusing on three main comparisons. First, we consider whether those who would accept and those who would refuse the vaccine differ with respect to key demographic measures, including age, gender, race and tenure of employment. We compare the means for a given characteristic among the vaccine acceptance and vaccine refusal groups and assess the statistical significance of each set of characteristics by regressing a binary measure of vaccine acceptance on a mutually exclusive and collectively exhaustive vector of dummy variables capturing all of the values of a particular characteristic. For example, to study whether a respondent’s race predicts vaccine acceptance, we regress the binary vaccine acceptance measure on an indicator variable for whether a respondent is Black and an indicator variable for whether a respondent is Hispanic, with White respondents forming the leave out group. We report the *p*-value from the regression F-test as a test of the joint significance of a given set of characteristics in predicting vaccine acceptance. We present analyses separately for corrections officers and non-security staff. To account for arbitrary heteroskedasticity in the regressors, regressions are estimated using robust (White) standard errors. 

Next, we consider whether those who accept versus refuse the vaccine differ with respect to their attitudes towards vaccines and medical care generally. For these analyses, we study whether the mean agreement scores for five measures of general vaccine acceptance differ according to an individual’s hesitancy to receive the COVID-19 vaccine. Statistical significance is determined using a series of *t*-tests. 

Finally, we consider how sources of trusted information differ according to race and vaccine acceptance status. We report the mean level of trust in a given group–for example, corrections officers or family members–separately for respondents of each race group (Non-Hispanic White, Non-Hispanic Black and Hispanic) and vaccine acceptance status. Statistical significance is determined by regressing trust in a given source of information on the full set of race indicators. Similar to our other regression analyses, we account for arbitrary heteroskedasticity in the regressors using robust (White) standard errors and test statistical significance using a regression F-test of the collective significance of the characteristics in predicting a respondent’s level of trust in a given source of information. We report the *p*-value from the regression F-test as a test of the joint significance of the predictor variables. 

## 4. Results

Regarding their willingness to be vaccinated, several differences emerged between the CO and non-security staff samples (Table 1). Overall, 73.5% (n = 1710) non-security personnel answered that they would accept a vaccine, compared to half (48.8%) of corrections officers (n = 589). 

Among the 1150 male non-security PADOC respondents surveyed, 77.9% (n = 896) would have accepted the vaccine; 50.7% (n = 495) of the 976 male CO respondents shared the same posture. We observed a similar pattern across female staff groups; 72% of non-security staff would accept the vaccine, compared to 42% of womxn COs surveyed.

We acknowledge that the PADOC personnel and inmate records measure two sex categories: male and female. We also acknowledge that those terms connote a restricted binary biological category, as opposed to one’s gender identity [76]. We further acknowledge that the terms “man” and “woman” are also limited in their descriptive capacities for gender-expansive and gender non-conforming individuals. Thus, we use the singular and plural term *womxn*, for example, which aligns itself with Black feminist and postcolonial theoretical expansions, as well as challenges the dichotomy of “man” and “woman”, and other incomplete fixed-response survey items, dichotomous measures of our social identities [77].

Among non-security staff, a larger proportion of vaccine refusers were younger than 40 years old (45.4%; n = 232), compared to their vaccine accepting counterparts (26.2%; n = 448). Among COs, 306 respondents younger than 40 years old represented 50.2% of the refusing subsample, compared to the 197 under 40 years old respondents who represented 33.4% of the vaccine accepting subsample. For neither personnel group did we observe statistically significant differences in vaccine refusal or acceptance according to self-identified race. Still, we present these comparisons to help situate a more nuanced understanding of how the CO population stands apart from the PADOC system-wide staff’s vaccine hesitancy reports.

Our measures of CO beliefs about vaccine effectiveness, overall health, and COVID-19 exposure risk differed significantly between officers who would accept the vaccine compared to those who would not (Table 2). Across five different belief measures, Likert response mean score differences in perception were strongly correlated with vaccine refusal or acceptance among prison security staff. On a scale of 1 to 5, where 5 indicates strong agreement with the survey item claim, COs who would refuse the vaccine reported less confidence in the ideas that vaccines are protective against diseases (2.80), that prison-based medical providers were trustworthy (2.29), and that COVID-19 vaccines were safe (1.94). COs who would accept the COVID-19 vaccine held more positive beliefs about the merits of vaccination (3.96), the credibility of prison-based health workers (3.47), and the integrity of the COVID-19 vaccines to come (3.39). Compared to COs who would refuse the vaccine, those who would accept it also reported a higher prevalence of personal vaccination history (3.35 vs. 4.19, respectively) and a stronger belief that they were at risk of COVID-19 exposure (3.37 vs. 3.87).

Last, we measured whether the mean scores for the COs’ trusted sources of vaccine information across both *Refuse* and *Accept* vaccination samples were consistent across racial groups (Table 3). Respondents ranked their trust in six different sources of truthful information about vaccines: medical professionals, government agencies, their corrections officer peers, PADOC leadership, family members, and trusted community leaders. Family members were the only trusted sources of information cited by CO respondents who would refuse the vaccine, and even then, their confidence was moderate for agreement scores that could range from 1 to 5. The differences in agreement that family members could be trusted sources of information did emerge across racial groups, but only slightly. Of officers who would refuse the vaccine, Black respondents offered the strongest agreement with the survey item claim that family members were trustworthy sources of information about vaccines (3.70). White respondents were slightly less confident in family member sources (3.14), and Hispanic officers were the least confident in their family members’ capacity to provide truthful information about vaccines (3.00). Within the *Accept* subsample of officers, we did not observe differences in trusted information source mean scores that varied across the three racial groups.

### Study Limitations

Given our sampling strategy and limitations, gleaning perspectives from prison staff working in womxn’s facilities is limited. The PADOC maintains operations for one womxn’s facility, SCI-Muncy, at which fewer than 600 full-time staff are employed (roughly 4 percent of the total PADOC staff sample) [78]. While it is not the case that all Muncy staff are womxn, all Muncy COs work exclusively with incarcerated womxn. It may follow that routine encounters with the on-site medical provision enterprise in a womxn’s prison facility may differentially shape beliefs that their employers are sufficiently and reliably equipped to serve as a truthful source of information regarding the COVID-19 vaccines, compared to the CO beliefs that are shaped by experiences unfolding in men’s prison facilities. To protect respondent anonymity, however, our analyses cannot include a descriptive summary of the SCI-Muncy staff responses. Still, we encourage continued research to not only measure correlations between staff gender from other facilities and their vaccine uptake willingness, but also any observable relationship between COs’ perception of vaccine administration, the gender of the incarcerated population in their charge, and the PADOC administration’s ideological and operational priorities for incarcerated men and womxn.

Concerning the analysis, we emphasize that the associations that we report between vaccine hesitancy and demographic and attitudinal measures are not being offered as evidence of a causal connection between these features and vaccine uptake. While age, gender, employment tenure, and attitudes towards vaccine uptake are all powerful and significant predictors of vaccine acceptance among corrections officers and non-security staff, each of these characteristics could itself be capturing the effects of an alternate set of underlying characteristics. For example, the race gap that we observe in vaccine acceptance could reflect any number of characteristics that differ, on average, between Black, White, and Hispanic respondents. These associations are thus not necessarily evidence of any particular channel through which an individual’s race predicts outcomes. However, the race gap remains an object of considerable social and policy interest, just as it is in many other areas of human and scientific inquiry. 

## 5. Discussion

These results reveal differences in vaccine acceptance and reported trust in sources of information about vaccines for adults who work within the Pennsylvania prison system. Corrections officers who reported an unwillingness to accept a COVID-19 vaccine placed less confidence in the purported health benefits of vaccination and were more skeptical about the credibility of varied sources of vaccine information. In reviewing COVID-19 vaccine uptake research to date, we find that our work follows certain empirical parallel arguments and diverges from others. In order to more responsibly interpret the results described above, we have begun to understand our study findings in tandem primarily with two conceptual research domains: legal epidemiology and situational and place-based medical distrust. 

### 5.1. Legal Epidemiology

Existing narratives of legal epidemiology center the utility of evaluating legal interventions as a mechanism for bridging normative public health law studies and empirical social scientific methods. Burris and colleagues [79] define legal epidemiology as “the scientific study and deployment of law as a factor in the cause, distribution, and prevention of disease and injury in a population” (p. 139). We contend that legal epidemiology explores how specific laws and legal practices impact health outcomes and conditions structural determinants of health. The public health research community has long embraced policy surveillance [79,80], or the means by which varied stakeholders use longitudinal legal and health data to “track the occurrence, antecedents, time course, geographic spread, consequences, and nature of disease, injury, and risk factors among the populations they serve” ([79], p. 141). 

Motivated by the study findings–and even its limitations–we aim to contribute to the legal epidemiology discourse by demonstrating that legal practices encompass an immensely wide array of behaviors, mandates, and decisions made by stakeholders who are neither lawyers nor judges. Prison staff operate with the authority of the state and thus can enforce, implement, and execute directives that go unchallenged. Regarding carceral sites and their organizational hierarchies, corrections officers, in the eyes of the incarcerated, represent the state and even these de facto delegates do not trust the government to ensure their welfare. There are perfectly legal practices unfolding in prisons to which these officers are bearing witness, and they are consciously opting out of employer-provided healthcare. These legal actors understand their own COVID-19 exposure, but refuse to take a vaccine that could stop the spread of a lethal disease in congregate detention settings, their workplace environments, and their communities beyond the prison walls.

### 5.2. Situational and Place-Based Medical Distrust 

An enduring challenge for communities that have limited and/or poor experiences with healthcare providers, is medical distrust, which again refers to the lack of confidence or trust in healthcare providers, medical institutions, or the healthcare system as a whole. Distrust can arise from certain negative experiences, such as medical errors, misdiagnosis, discrimination, or unethical practices, which can lead to feelings of frustration, fear, anger, and disillusionment among patients. Medical distrust particularly affects vulnerable and marginalized communities, such as people of color or those with lower socio-economic status who may have experienced historical or ongoing systemic injustices in the healthcare system, especially routine providers’ racial biases and discrimination [81]. 

Doubts about contemporary prison-based health provider competency [58,82,83,84,85] does not help efforts to correct these legacies, either, and this eroded trust is particularly pronounced among both incarcerated people [86] and the corrections officers [87,88] charged with their care. Some evidence suggests that the *values* dimension of healthcare distrust held by people who doubt their provider’s offer of respect, honesty, caring, dependability, and confidentiality, is less of an individual determinant of outcomes than personal health status and access to healthcare [52]. Shea and colleagues [89] assert that the *values* dimension of healthcare provision mandates provider respect, honesty, care, dependability, and confidentiality, while the *competence* dimension of care concerns the technical skill capacity needed for effective diagnosis and treatment. We agree with others [90] that demonstrable competence must also include sound communication skills, bedside manner, gathering accurate medical histories, and providing information for effective treatment. Among COs that would refuse the COVID-19 vaccine in our study, respondents offered little endorsement of vaccine safety or necessity, despite their admissions of perceived exposure risk (see Table 2). Simply put, when asked to report their agreement with the claim, “you trust the medical providers here”, these respondents held little (mean score of 2.29 on a scale of strong disagreement, 1, to strong agreement, 5). Officers who would accept the vaccine, however, did agree with the claim (mean score of 3.47), which suggests that COs are not uniformly distrustful of the PADOC healthcare providers or public health officials more broadly. Rather, it is officers who would refuse the vaccine who do not trust the providers at their place of employment. To protect respondent anonymity, our analyses do not disaggregate responses to the facility, but continued research should examine how COs’ occupational health beliefs vary across workplace sites. 

These challenges are not restricted to carceral sites, however. For example, Cafferty and colleagues studied medical communication practices in Augusta-Richmond County, Georgia and leveraged The People and Places Framework for health communication [91] to identify which attributes of patients’ place threaten community trust in public health and medical recommendations. Interview data collected from a majority-Black sample suggest that four-local level attributes of place threaten county residents’ medical trust: access to products and services (e.g., fresh food, timely affordable and quality healthcare), social structures (unreliable police response, neighborhood blight and neglect), physical structures (diminishing housing stock and social disregard cast by medical staff who work in the neighborhood but do not engage with its residents), and cultural and media messages (a justified fear of public health messaging from the same government that preyed on their community for decades). Their findings reveal a much broader, but still local “web of services, policies, and institutions, beyond interactions with health care, that influence the trust placed in health officials and institutions” ([92], p. 6). These root causes can be addressed and outcomes that follow corrective efforts will likely be positive. 

In scenarios where one’s belief about place-based safety in their physical environment has steered their trust in healthcare providers and larger institutions, effective COVID-19 safety communication designed and disseminated by sources that patients trust, are promising. Different healthcare communication methods can be used, such as verbal communication, written communication, electronic communication, and even prison-based peer-to-peer healthcare mentorship [93]. Particularly since our study respondents did report more trustful relationships with non-prison affiliated sources of information about the COVID-19 vaccine, we urge prison systems to support communications created by parties external to the system’s leaders, policies, and practices. Again, among the COs who would refuse the vaccine, Black respondents offered the strongest agreement with the survey item claim that family members were trustworthy sources of information about vaccines (3.70). White respondents were slightly less confident in family member sources (3.14), and Hispanic officers were the least confident in their family members’ capacity to provide truthful information about vaccines (3.00). Still, in order to communicate with marginalized groups and/or those that have experienced harm in healthcare settings (including dismissal and inaction on the part providers) it is essential for healthcare providers to be culturally sensitive and use appropriate language to ensure effective and relatable communication with patients and their families from diverse backgrounds and varied structures [94,95,96]. Community-based organizations that expressly attend to these very challenges are better prepared and require material support to effectively reach and protect vulnerable people who still respect and believe their familial and local messengers [97].

## 6. Conclusions

Our findings surface additional questions and possibilities for deeper research across disciplinary traditions and analytic frameworks. We are especially interested in readers using the study data to broaden our understanding of prison workers’ (un)conscious engagement with medical racism, occupational health, and structural competency. 

### 6.1. Medical Racism

Medical racism refers to systemic discrimination against individuals and communities of different races in healthcare settings. This phenomenon has had a long history, with minoritized ethnoracial groups across socioeconomic class ranks suffering from misdiagnosis, inadequate treatment, and a general lack of compassion and understanding from healthcare providers [98,99]. There have been numerous documented patterns of medical racism spanning the history of the US, where doctors discriminated against Black [100,101], Native or Indigenous [4], Latinx [102], womxn of color [103], children of color [104,105], poor [106], queer or gender non-conforming [107], and disabled [6] patients of color, leading to poorer health outcomes for these groups and even death. Efforts are being made to address this issue, but it remains a significant challenge that needs continued attention and action.

The observed differences in beliefs about the vaccine and who officers identified as a trusted source of information about the vaccine were not as pronounced across the racial groups as we had anticipated. Given the existing scholarly literatures exploring the enduring medical racism confronted by Black, Indigenous, and Latinx people in the US, at times conducted most lethally within prison sites [41], we expected to see clearer racial delineations in vaccine acceptance. Specifically, and based on prior work, we hypothesized that the relative proportion of Black COs who would refuse the vaccine would exceed the relative proportions of their White counterparts. Our findings do not support that assumption, a finding that should be considered and explored in future work.

Similarly, officers who would refuse the COVID-19 vaccine place moderate to scant trust in the most media prevalent and state-promoted sources of vaccine information available: guidance from the US Centers for Disease Control [108,109]. For this sample, a pervasive crisis of medical distrust in government transcends racial group membership. Thus, future research should examine how (mis)information is uniquely disseminated within incarcerated and CO racial groups, which could shed light on how risk perceptions of exposure to COVID-19 or its vaccines, are grounded, operationalized, and generally racialized by adults who live and work in prisons.

### 6.2. First Responders’ Occupational Health 

Occupational health focuses on the physical and psychological wellbeing of workers in their workplace environment. It involves identifying, evaluating, and controlling workplace health hazards that may cause harm to workers, as well as promoting the overall health and wellness of employees through various programs, policies, and initiatives. The goal is to prevent workplace injuries, illnesses, and deaths, and to optimize the physical and mental health of workers so that they may perform their duties safely and effectively. First responder occupational health focuses specifically on the health and wellbeing of firefighters, police officers, emergency medical technicians, and more recently jail and prison staff. These professionals are often exposed to hazardous conditions, physical demands, and emotional stress that can negatively impact their health over time [110]. Given their routine exposure to harm, these professionals need to receive regular medical evaluations, health screenings, and specialized care to prevent and manage these conditions. 

Fulfilling these goals generally requires worker willingness to discuss their health status and goals with their employer. Despite their exposure to dangers and poor health outcomes [111,112], our CO respondents, demonstrate a lack of trust in their employer’s capacity to offer truthful information about their healthcare risks or the interventions leveraged to allegedly mitigate them. Thus, we believe that there is much to be done regarding healthcare ambassadorship and identifying methods for effectively educating workers who are distrustful of their employers and the professional medical enterprise, writ large. 

### 6.3. Structural Competency

Structural competency is an institutional and individual capacity to recognize and understand how social, economic, and political structures shape people’s health and well-being [113,114]. It involves examining the larger contexts and systems that influence individual health outcomes, rather than just focusing on patients’ or clients’ individual behaviors or choices. The concept of structural competency is important in healthcare and medicine because it acknowledges social determinants of health and highlights the need to address underlying structural inequities in order to promote health equity and better health outcomes for all individuals. Some prison systems are working to develop and incorporate these competencies in their policy and operational design, but much of the research studying these shifts is focused on how providers hold the totality of a detainee’s history and experiences during a medical encounter, offering diagnoses, and referral to treatment (or not) [58,115]. The experiences of jail, prison, and detention security staff, particularly since their health is routinely at risk by virtue of their duties, merit a deeper and more comprehensive analysis from the public health, medical, sociolegal research communities, and other stakeholders working to expand a discourse that will meet the challenges of deploying legal authority for good. 

## Figures and Tables

**Table 1:** Demographic characteristics of the PADOC Staff Sample (n = 3533).

Non-Security Staff (n = 2325)				Corrections Officers (n = 1208)		
	Refuse	Accept	*p*	Refuse	Accept	*p*
N	571	1710		609	589	
Sex (%)			<0.01			0.03
Male	254 (44.6)	896 (52.5)		481 (79.8)	495 (84.5)	
Female	315 (55.4)	810 (47.5)		122 (20.2)	91 (15.5)	
Race (%)			0.37			0.11
White	498 (87.2)	1547 (90.5)		529 (86.9)	519 (88.1)	
Black	22 (3.9)	91 (5.3)		23 (3.8)	39 (6.6)	
Hispanic	12 (2.1)	30 (1.8)		13 (2.1)	14 (2.4)	
Age (%)			<0.01			<0.01
18–24	10 (1.8)	6 (0.4)		27 (4.5)	9 (1.5)	
25–29	57 (10.0)	81 (4.7)		89 (14.7)	41 (7.0)	
30–39	165 (29.0)	361 (21.1)		190 (31.4)	147 (25.0)	
40–49	198 (34.8)	527 (30.8)		206 (34.0)	203 (34.6)	
50–64	131 (23.0)	657 (38.4)		91 (15.0)	178 (30.3)	
65+	8 (1.4)	77 (4.5)		3 (0.5)	9 (1.5)	
Employment Tenure (%)			<0.01			<0.01
<1 year	49 (11.1)	99 (6.9)		31 (6.7)	36 (7.4)	
2–4 yrs	120 (27.3)	319 (22.3)		104 (22.6)	91 (18.8)	
5–9 yrs	81 (18.4)	338 (23.7)		120 (26.0)	101 (20.9)	
10–14 yrs	78 (17.7)	201 (14.1)		82 (17.8)	95 (19.6)	
15–24 yrs	100 (22.7)	352 (24.7)		113 (24.5)	139 (28.7)	
25+ yrs	12 (2.7)	119 (8.3)		11 (2.4)	22 (4.6)	

*p*-value obtained from an F-test, in which the null hypothesis is that a given variable does not predict refusal or acceptance of the vaccine. Percentages do not always add up to 100 due to rounding estimates and missing cases. Data are from a survey of corrections staff in the Pennsylvania Department of Corrections carried out in January and February 2021.

**Table 2:** Beliefs about vaccine effectiveness, overall health, and COVID-19 exposure risk: stratified by whether participants would take the COVID-19 vaccine if made available to them for free.

	Corrections Officers		
	Refuse	Accept	*p*
Vaccines keep you from getting diseases.	2.80	3.96	<0.01
As an adult, you were vaccinated against at least one disease.	3.35	4.19	<0.01
You trust the medical providers here.	2.29	3.47	<0.01
COVID-19 Exposure Risk	3.37	3.87	<0.01
Beliefs about COVID vaccine safety	1.94	3.39	<0.01

*p*-value obtained from a *t*-test of differences in means. Data are from a survey of corrections staff in the Pennsylvania Department of Corrections carried out in January and February 2021. Items are based on questions assessing who participants trust would tell them the truth about vaccines. Cells include averages on the 5-item Likert type scales as well as several Yes/No questions. For the Likert scale questions, higher scores reflect more trust; for the binary questions, higher scores reflect more perceived contact with the COVID-19 virus.

**Table 3:** Trust in sources of truth about vaccines: stratified by race and opinion about whether participants would take COVID-19 vaccine if made available to them for free.

	Refuse				Accept			
	Black	White	Hispanic	*p*	Black	White	Hispanic	*p*
Medical professionals	3.00	2.55	2.69	0.11	4.00	4.10	4.14	0.83
Government	1.78	1.51	1.69	0.29	3.21	3.04	3.21	0.65
Other staff	2.39	2.28	2.00	0.55	2.87	3.08	3.43	0.29
PADOC Leadership	1.87	1.82	1.69	0.87	3.26	3.13	3.64	0.17
Family	3.70	3.14	3.00	0.03	3.97	3.84	4.00	0.48
Community leaders	2.57	2.29	2.15	0.37	3.38	3.27	3.50	0.55
N	23	529	13		39	519	14	

*p*-value obtained from an F-test, in which the null hypothesis is that a respondent’s racial group ascription does not predict which sources of information about the COVID-19 vaccine are most trusted. F-tests are derived from a linear regression analysis of trust in a given institution or group of people on a vector of race/ethnicity dummies, with regressions run separately for those who would accept and those who would refuse the vaccine. Data are from a survey of corrections staff in the Pennsylvania Department of Corrections carried out in January and February 2021. Items are based on questions assessing who participants trust would tell them the truth about vaccines. Cells report averages on the 5-item Likert scale.

## Data Availability

Not applicable.
